# Differences in biochemical markers between Heart-transplanted and Left Ventricular Assist Device implanted patients, during cardiac rehabilitation

**DOI:** 10.1038/s41598-018-29193-0

**Published:** 2018-07-17

**Authors:** Vittorio Racca, Paolo Castiglioni, Claudia Panzarino, Marina Saresella, Ivana Marventano, Alessandro Verde, Fabrizio Oliva, Maurizio Ferratini

**Affiliations:** 1 Santa Maria Nascente Institute IRCCS, Don Gnocchi Foundation, Cardiology Rehabilitation Department, Milan, 20148 Italy; 2 Santa Maria Nascente Institute IRCCS, Don Gnocchi Foundation, Biomedical Technology Department, Milan, 20148 Italy; 3 Santa Maria Nascente Institute IRCCS, Don Gnocchi Foundation, Laboratory of Molecular Medicine and Biotechnology, Milan, 20148 Italy; 4 Niguarda Hospital, De Gasperis Cardio Center, Milan, 20162 Italy

## Abstract

Heart transplant (HTx) and left ventricular assist device (LVAD) implant are the best options for symptomatic end stage heart failure, but LVAD patients show lower rehabilitative outcome than HTx patients. To investigate the causes, we compared biomarkers levels and their association with rehabilitative outcome in 51 HTx and in 46 LVAD patients entering the same cardiac rehabilitation program. In both groups, routine biomarkers were measured at start (T1) and end (T2) of cardiac rehabilitation while homocysteine, leptine and IGF-1 were measured at T1 only. HTx patients had lower lymphocyte, platelets, glucose, total proteins and albumin at T1; differences with LVAD patients vanished during rehabilitation when new cases of diabetes were observed in HTx. By contrast, total cholesterol, LDL and HDL fractions, leptin and IGF-1 were higher in HTx patients. The increase from T1 to T2 in six-minute walking test distance, measure of functional rehabilitation outcome, was positively associated with homocysteine and IGF-1 levels in HTx patients. In conclusion, during rehabilitation care should be paid to the early occurrence of dyslipidemia and hyperglycemia in HTx patients, which also require a proper protein dietary support. IGF-1, dangerously low in LVAD patients, might contribute to their lower rehabilitative outcome.

## Introduction

Heart failure affects 1–2% of the adult population in developed countries, with high morbidity and mortality at the most advanced stage of severity. When end-stage heart failure patients are symptomatic despite optimal medical therapy, Heart Transplantation (HTx) is the best option in terms of survival or quality of life. However, HTx is contraindicated over 70 years of age and many patients die before a suitable heart becomes available. An alternative option is the long-term mechanical circulatory support, and the implant of a left ventricular assist device (LVAD) is always more extensively implemented, given the good results of clinical trials^1^.

Cardiac rehabilitation improves functional capacity and health status both for patients after HTx or LVAD implant and its feasibility, safety and potential for benefit have been documented^2^. However, we recently showed a lower rehabilitative outcome in LVAD implanted patients than in HTx patients, even after correction for demographic and anthropometrics characteristics and for the duration of rehabilitation^3^. This suggests that other factors may be responsible for the better rehabilitative outcome in HTx patients. Understanding these mechanisms might help to better target the rehabilitation program according to the type of surgery, possibly improving the rehabilitative outcome.

Main aim of this study is to highlight the possible differences in hematological and biochemical markers between HTx and LVAD patients entering a cardiac rehabilitation period few days after surgery; secondary aim is to identify possible associations between the levels of the investigated markers and the rehabilitative outcome. In perspective, this type of information might help to improve the best clinical approach in LVAD and HTx patients, tailoring the rehabilitation program to their needs

## Methods

This open-label observational study was carried out at the Cardiology Rehabilitation Department of the Don Gnocchi Foundation’s Santa Maria Nascente Institute in Milan, Italy, according to the principles of the Helsinki Declaration and Good Clinical Practice and in observance of anti-discrimination regulations and standard privacy procedures. The protocol was approved by the Institute’s Ethics Committee. The study was performed after all the patients involved gave their written informed consent.

We considered the 97 adult patients of our previous study^3^, consecutively admitted to the Cardiac Rehabilitation Unit of our Institute as in-patients after HTx (n = 51) or LVAD implant (n = 46) along a period of two years. All patients were Caucasians admitted as in-patients the day of the discharge from the transplant Centre located in Milan. No exclusion criteria was established with the exception of refusing the consent.

### Patients characteristics

Differences between groups in demographic and anthropometric characteristics are reported in Table 1 and can be summarized as greater prevalence of males, higher age and body mass index in LVAD than in HTx patients (see more details in^3^). The groups also differed for the underlying heart diseases, being dilated cardiomyopathy more frequent in HTx patients, and post-ischemic cardiomyopathy more frequent in LVAD patients. Implanted LVAD were the Heartmate II device (Thoratec Corporation, Pleasanton, California) and the Heartware system (HeartWare Inc., Framingham, Massachusetts).

**Table 1:** General characteristics, type of heart disease and drug therapy at admission as mean (SD) or number of cases (percentage), with p the statistical significance of the difference between percentages in the groups

	LVAD Patients (N = 46)	HTx Patients (N = 51)	p
Male Sex	41 (89%)	30 (59%)	<0.01
Age (yrs)	57.3 (7.8)	48.0 (13.6)	<0.01
Body Mass Index (kg/m^2^)	25.3 (4.0)	22.2 (3.8)	<0.001
Time from Surgery (days)	34.6 (16.8)	27.7 (14.9)	<0.05
***Type of Cardiomyopathy***			
Dilated	19 (41%)	35 (69%)	<0.01
Post-ischemic	26 (57%)	5 (10%)	<0.01
Valvular	1 (2%)	2 (4%)	0.99
Postchemotherapy	0 (0%)	3 (6%)	0.24
Hypertrophic/Arrythmogenic	0 (0%)	6 (12%)	0.03
***Drug Therapy***			
Beta blockers	31 (67.4%)	19 (37.2%)	<0.01
Loop Diuretics	31 (67.4%)	25 (49%)	0.10
ACE Inhibitors	31 (67.4%)	32 (62.7%)	0.67
AT1-receptor blockers (ARB)	3 (6.5%)	2 (3.92%)	0.67
Aldosterone antagonists (MRA)	18 (39.1%)	1 (2.0%)	<0.0001
Ca-Channel Blockers	2 (4.3%)	9 (17.6%)	0.05
Transdermal Nitrates	1 (2.2%)	0 (0.0%)	0.47
Acetylsalicilic Acid	43 (93.5%)	42 (82.4%)	0.13
Other Antiplatet drug	44 (95.7%)	0 (0.0%)	<0.001
Oral Anticoagulants (VKA)	46 (100%)	3 (5.9%)	<0.001
Amiodarone	21 (46.6%)	1 (2.0%)	<0.001
Cardiac Glycosides (Digoxin)	4 (8.7%)	0 (0.0%)	<0.05
Statins	9 (19.6%)	4 (7.8%)	0.14
Allopurinol	4 (8.7%)	2 (3.9%)	0.42
Proton Pump Inhibitors	38 (82.6%)	48 (94.1%)	0.11
Tiroxine	7 (15.2%)	15 (29.4%)	0.14
Benzodiazepines	12 (26.1%)	14 (27.5%)	0.99
Insulin	7 (15.2%)	8 (15.7%)	0.99
Oral Antidiabetics	3 (6.5%)	0 (0.0%)	0.10
Corticosteroids	1 (2.2%)	51 (100%)	<0.001
Cyclosporine or Tacrolimus	0 (0.0%)	51 (100%)	<0.001
Mycophenolate	0 (0.0%)	50 (98.0%)	<0.001

p after Mann Whitney U test or Exact Fisher test.

### Diet, Drugs Therapy and Rehabilitation

During the study period, all the patients received a standard diet with controlled caloric support (30 Kcal/kg/day), delivering 20% dietary proteins, 30% fat, and 50% carbohydrates. The diet included fruits and vegetables. Dietary intake was checked by nurses.

All drugs taken during the study are reported in Table 1. In the HTx group the immunosuppressive treatment consisted of prednisolone combined with cyclosporine or tacrolimus, and mycophenolate mofetil. The doses were adjusted according to the laboratory results and endomyocardial biopsies reports. According to the guidelines of the International Society for Heart Lung Transplantation (ISHLT), allograft rejection was diagnosed using the standardized grading system^4^. In the LVAD group the antithrombotic treatment included the combination of double antiplatelet therapy and oral vitamin K antagonist anticoagulants, in accordance with international guidelines^5^.

Both groups attended the same rehabilitation program. The program, which planned duration of at least 3 weeks, was continued throughout the period of hospitalization until discharge. The standardized and supervised rehabilitation program was performed in accordance with the international guidelines^6^. It consisted of incentive spirometry, breathing exercises, sub-maximal gradually incremental endurance training at least 30 minutes/day, walking on treadmill or cycling on cyclette, as previously described^3^. Muscular activities included limbs flexion, extension and abduction, neck flexion and extension, trunk flexion, extension and rotation, performed with aerobic exercises. When necessary, a pointed physical therapy was focused on improving balance to obtain independent functional mobility.

### Measures of Functional Capacity and Biochemical Markers

The six-minute walk test was performed to measure the functional exercise capacity, in accordance with the guidelines of the American Thoracic Society^7^. The test provided the distance walked in six minutes that we expressed as absolute distance in meters (6MWD) and as percentage of the predicted distance for an individual of the same sex, weight and height (6MWD%), according to the statistical model described in^8^. The test was repeated twice: upon study entry (T1) and the day of discharge (T2). The rehabilitative outcome was quantified as increase of 6MWD% from T1 to T2. The score of the Barthel scale was recorded at T1 and at T2 as measure of performance in daily-life activities.

Routine chemistry tests on urine and venous samples by UniCel DxC 800 Synchron (Beckman Coulter; Brea, California) included measures of Creatinine, Glucose, Total Protein, Albumin, Cholesterol, Cholesterol HDL, Triglycerides, Vitamin B12, C-Reactive Protein, Folic Acid, Alanine Aminotransferase, Aspartate Aminotransferase, Gamma Glutamyl Transferase, Total Bilirubin and Homocysteine. The blood sample for homocysteine was kept on ice until the analysis. Red and white blood cells were counted by Sysmex XE-2100 (Dasit; Milan, Italy). Blood samples were centrifuged at 3000 rpm for 10 minutes to obtain serum or plasma that were frozen and stored at −70 °C. Serum concentrations of Leptin and Insulin-like Growth Factor 1 (IGF-1) were determined by ELISA (Quantikine Immunoassay; R&D Systems, Minneapolis) with minimum detectable dose of 7.8 pg/ml for Leptin and 0.026 ng/ml for IGF-1.

The clinical protocol included the collection of a spot urine and venous sample for the above measurements at T1 and at T2, with exclusion of Homcysteine, Leptine and IGF-1, which were measured only at T1, for the whole group of 97 patients. However, due to a procedural change in the way patients data were transcribed in their clinical records, measures of few biomarkers were available on a lower sample of patients. These markers were: Leptin and IGF-1, available on 63 of 97 patients; vitamin B12 and Folate, available on 55 patients; Homocysteine and Ferritin respectively available on 52 and 50 patients.

### Statistical Analysis

LVAD and HTx groups were compared by two-tailed Mann Whitney U test at T1 and at T2 separately, with threshold for statistical significance at p < 0.05. To identify a statistical association between any of the markers measured at T1 and the outcome of the rehabilitation program, the Kendall’s tau correlation coefficient was calculated between each biomarker and the increase in 6MWD%. Positive coefficients mean that greater values of the marker at T1 are associated with higher functional increments at the end of rehabilitation.

### Data availability

The datasets generated and analyzed during the current study are available from the corresponding author on reasonable request.

## Results

Admission to cardiac rehabilitation occurred 42 ± 44 (LVAD) and 28 ± 15 (HTx) days after surgery; discharge occurred 35 ± 16 (LVAD) and 40 ± 17 (HTx) days after admission. Three HTx (5.9%) and twelve LVAD (26.1%) patients were diabetic at T1. The number of HTx diabetic patients increased to six at T2. Cardiac rehabilitation was interrupted for less than two weeks in two HTx patients due to graft rejection episodes. Four LVAD patients had complications (n = 3 ventricular tachycardia, n = 1 severe anemia) interfering with the rehabilitative programme or leading to its temporary interruption.

### Differences in biomarkers levels

**Figure 1:**
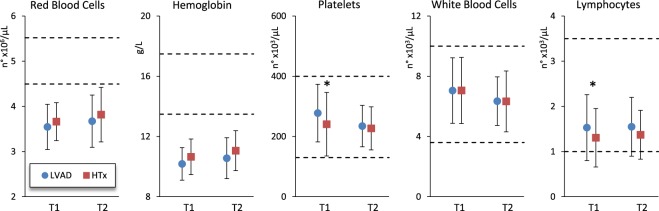
Hematological markers in LVAD and HTx groups at the beginning (T1) and end of rehabilitation (T2): mean values and SD. The “*” indicates significant differences (P < 0.05) between T1 and T2. The horizontal dashed lines indicate lower and upper normal limits.

Anaemia, indicated by mean values of red blood cells count and haemoglobin lower than the normality range, was detectable both at T1 and T2, without significant differences between LVAD and HTx groups (Fig. 1). Platelets, white blood cells count and lymphocytes mean values were normal, but HTx patients had significantly lower lymphocyte and platelets counts than LVAD patients at T1 (Fig. 1).

**Figure 2:**
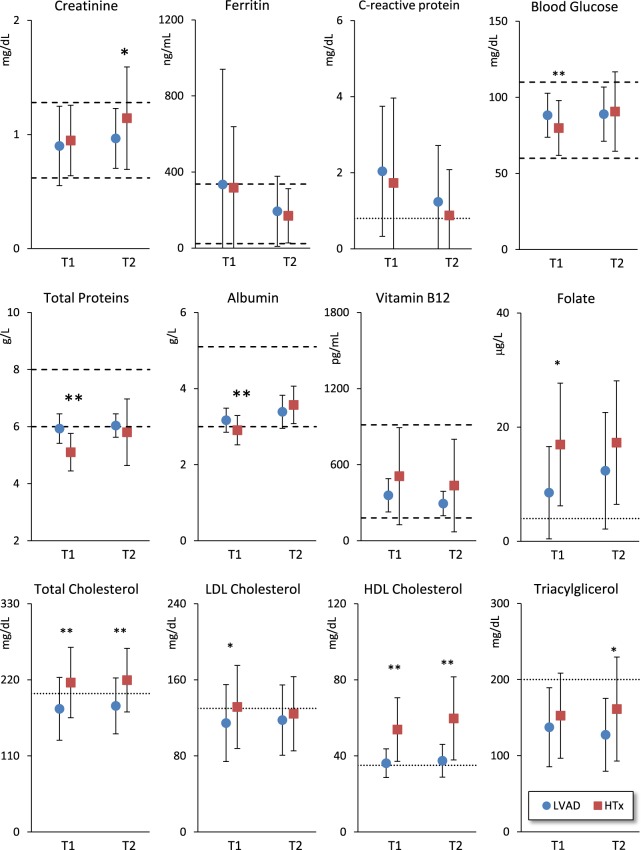
Renal function, inflammatory and nutritional markers at T1 and T2 (see Fig. 1 for symbols). The single horizontal dotted line indicates the risk limit threshold.

Renal function was normal (Fig. 2). However, while creatinine plasma level was similar in the two groups at T1, it was greater in HTx patients at T2. Inflammatory indices, i.e. C-reactive protein and Ferritin, were elevated at T1 and tended to reach normal values at T2, without differences between groups (Fig. 2).

Regarding metabolic bio-markers (Fig. 2), blood glucose, total proteins and albumin were lower in HTx patients at T1 but not at T2, whereas total cholesterol, LDL and HDL fractions were higher in HTx patients at T1, the differences remaining significant for total and HDL cholesterol at T2. Conversely, triacylglycerol levels, similar in LVAD and HTx patients and within the normal range at T1, were greater in LVAD patients at T2. Mean folate and Vitamin B12 were within the normal range in both group, but folate was lower in LVAD patients.

**Figure 3:**
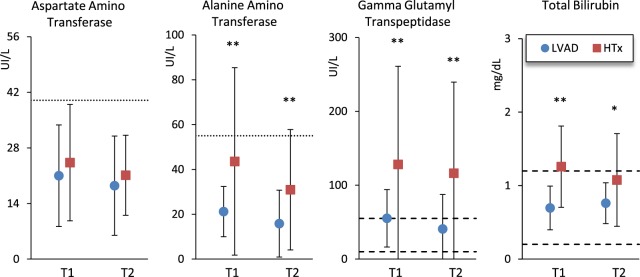
Biomarkers related to liver function and total bilirubin in LVAD and HTx groups at T1 and T2 (see Fig. 2 for symbols).

Figure 3 shows biomarkers related to liver function and bilirubin. Mean levels of aspartate amino transferase were within the normal range without differences between groups, whereas HTx patients had mean alanine amino-transferase, gamma glutamyl-transpeptidase and bilirubin levels significantly greater than LVAD patients, both at T1 and at T2.

**Figure 4:**
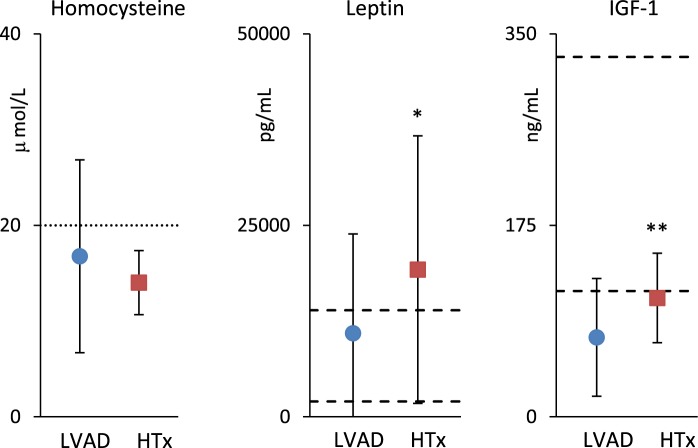
Plasma levels of Homocysteine, Leptin and IGF-1 measured at T1: mean and SD. Normal values are between the dashed lines (Leptine and IGF-1); the risk limit is above the dotted line (homocysteine). The “*” and “**” indicate significant differences between LVAD and HTx at P < 5% and P < 1%.

As to biomarkers measured at T1 only (Fig. 4), mean homocysteine levels did not differ between groups, although values among LVAD patients had larger dispersion around the mean. Plasma leptin and IGF-1 levels were higher in HTx patients.

### Relationship between measures at T1 and rehabilitative outcome

The distance walked in the six-minute walking test was similar at T1: 254 ± 116 meters in HTx patients, 241 ± 94 meters in LVAD patients, respectively corresponding to 40.9% ± 17.6% and to 42.4% ± 16.8% of the predicted value for an healthy individual of the same anthropometric characteristics. At T2, 6MWD increased by 177 ± 100 m in the HTx group and by 129 ± 114 m in the LVAD group, respectively reaching 69.8% ± 14.5% and 64.7% ± 15.6% of the predicted value, with greater rehabilitative outcome, quantified as increment of 6MWD%, for the HTx (+28.8%) than the LVAD (+22.3%, p < 0.05) group. The Barthel score was similar at T1, being 80 (80–85) points, as median (first-third quartile), both in HTx and LVAD patients. At T2, the score reached 100 (100–100) in HTx patients and 100 (95–100) in LVAD patients. The improvement of Barthel score after rehabilitation was +15 (10–20) points in LVAD patients, and slightly greater, +20 (15–20) points in HTx patients (p < 0.07).

**Figure 5:**
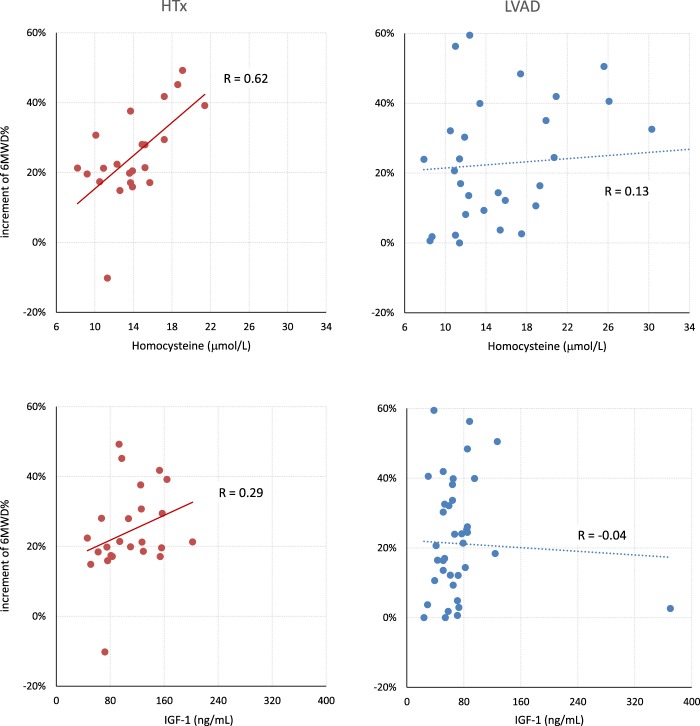
Increase in 6MWD% after rehabilitation, vs. homocysteine levels (upper panels) and IGF-1 (lower panels) measured at T1, in HTx and LVAD groups separately.

Table 2 reports the Kendall’s correlations between increments in 6MWD% and each biomarker at the start of the rehabilitation program. Increments in 6MWD% were associated with homocysteine level (p < 0.05) in HTx patients only (see also Fig. 5). The tau coefficient greater than zero indicates that higher is the biomarker level, greater is the rehabilitative outcome. A positive association with IGF-1 (p = 0.05) was also present in HTx patients.

**Table 2:** Kendall’s Tau correlation coefficient between each biomarker and increment in 6MWD%, with statistical significance p.

	LVAD Patients		HTx Patients	
	Tau	p	Tau	p
Red Blood Cell	−0.007	n.s.	−0.006	n.s.
Hemoglobin	0.055	n.s.	−0.111	n.s.
Platelets	0.087	n.s.	−0.004	n.s.
White Blood Cell	0.144	n.s.	−0.054	n.s.
Lymphocytes	0.005	n.s.	−0.076	n.s.
Creatinine	−0.062	n.s.	−0.039	n.s.
Ferritin	−0.095	n.s.	0.047	n.s.
C-reactive protein	0.172	0.09	0.094	n.s.
Blood Glucose	−0.033	n.s.	0.144	n.s.
Total Proteins	−0.120	n.s.	−0.115	n.s.
Albumin	−0.186	0.08	−0.135	n.s.
Vitamin B12	0.046	n.s.	0.034	n.s.
Folate	−0.079	n.s.	−0.080	n.s.
Total Cholesterol	0.108	n.s.	−0.045	n.s.
LDL Cholesterol	0.124	n.s.	−0.046	n.s.
HDL Cholesterol	0.039	n.s.	−0.055	n.s.
Triacylglicerol	0.104	n.s.	0.039	n.s.
Aspartate Amino Transferase	0.010	n.s.	0.034	n.s.
Alanine Amino Transferase	−0.015	n.s.	−0.129	n.s.
Gamma Glutamyl Transpeptidase	0.076	n.s.	0.105	n.s.
Total Bilirubin	−0.023	n.s.	−0.117	n.s.
Homocysteine	0.212	n.s.	0.366	0.02
Leptin	0.048	n.s.	0.143	n.s.
IGF-1	0.108	n.s.	0.283	0.05

p values greater than 0.10 are reported as not significant, n.s.

## Discussion

To the best of our knowledge, this is the first study comparing biochemical makers at admission of cardiovascular rehabilitation and their association with the improvements in rehabilitative outcome in heart failure patients treated with HTx or LVAD. We found some substantial differences between the two groups in inflammatory, haematological and nutritional markers: these differences only in part decreased after rehabilitation. We also found some associations between the final rehabilitative outcome and the biomarker levels before rehabilitation.

Anemia and elevation of inflammation markers at T1 are common findings after heart surgery. However, it is unexpected that biomarkers of anaemia are similar in the two groups at T1 given the high frequency of haemolysis caused by the LVAD centrifugal pump. This is probably due to the recent technical improvements of LVAD devices. The lower lymphocyte count in HTx patients at T1 could be consequence of their immune-suppressive treatment, and the progressive reduction of immune-suppressive medications during rehabilitation may explain the minor difference between groups at T2. Also platelets count was lower in HTx patients at T1 but not at T2. This could be consequence of a slight decrease of platelets count in LVAD patients, due to their larger consume of low molecular weight heparin and other antithrombotic drugs to prevent pump thrombosis and stroke, or consequence of an increased breakdown of circulating platelets induced by shear stress and trauma caused by the rotor pump.

Total proteins and albumin lower in HTx patients (Fig. 2) confirm their poor nutritional status at baseline, as previously shown by anthropometric data^3^. The accurate monitoring of protein dietary intake during rehabilitation may have favoured an increase of protein levels in the HTx group, so that the difference between groups vanished at T2. This is clinically important because hypoalbuminemia is associated with poor outcome in advanced heart failure^9^. Probably because of the proper dietary intake administered during rehabilitation, mean levels of vitamin B12 were normal, without any signs of the decrease previously reported at the end of rehabilitation in heart surgery patients^10^.

Folate levels were higher in HTx patients at T1, while homocysteine concentrations were similar in both goups, but with high standard deviation in LVAD patients due to very high levels in some patients. Hyper-homocysteinemia is a cardiovascular risk factor involved in the pathogenesis of atherosclerosis^11^ and ischemic heart disease was recorded as more prevalent in the LVAD group.

IGF-1 levels were lower than the normality range in LVAD patients. This is a potentially harmful condition that may increase mortality^12^. IGF-1 levels were higher in the HTx group and the higher levels may have provided anabolic and cellular proliferative stimuli, which likely favoured the improvement of nutritional markers and skeletal muscle mass increase, positively contributing to the rehabilitation process. Leptin levels, higher in HTx patients, generally reflect body fat stores, being the adipose tissue the major source of circulating leptin. However, this cannot be the cause of their higher levels in our HTx patients, because both body mass index and body fat stores were higher in LVAD patients^3^. More likely, the prednisone treatment in the HTx group might have had an effect on leptin levels elevation because corticosteroids may stimulate leptin release from adipocytes^13^.

The plasma lipids at T1 were higher in the HTx group probably because the immunosuppressive therapy with cyclosporine or tacrolimus induces hypercholesterolemia as side effect of calcineurin inhibitors^14^. More patients were treated with statins in the LVAD (19.6%) than in the HTx (7.8%) group, but the groups differed also considering only patients not treated with statins. After rehabilitation the difference between groups in LDL was no more significant, likely because of the beneficial effect of aerobic exercises performed during rehabilitation^15^. These results show that hypercholesterolemia tends to occur in HTx patients within one month after surgery, and physicians should take care of this clinically relevant issue since the beginning of cardiac rehabilitation. This is a challenging problem, considering that in HTx patients the diet is difficult to establish and has scarce influence on cholesterolemia, and that the treatment with statins is delicate because of the high incidence of myositis and rabdomyolysis^16^.

Diabetes increases the risk of infections and vascular complications^15^. The prevalence of diabetes mellitus was high in LVAD patients (26%) and the mean glucose plasma levels higher than in HTx patients. However, we also found a high incidence of new cases of diabetes mellitus in HTx after rehabilitation, so that at T2 the prevalence of diabetes was relatively high also in the HTx group (12%) and the difference vs. LVAD patients in glucose levels no more significant. The increase of blood glucose after rehabilitation in the HTx group is likely due to the prednisone therapy as part of immunosuppressive treatment. These findings warn cardiologists suggesting the need of immediate preventive dietary measures and strict blood glucose monitor in HTx patients receiving immunosuppressive treatment. HTx patients also had liver enzymes and bilirubin higher than LVAD patients, suggesting a possible hepatotoxic effect induced by immune-suppressive medications.

Homocysteine levels at T1 were associated with 6MWD% increments in HTx patients. The positive correlation coefficient indicates that higher was the biomarker at T1, larger was the functional increment. Patients with elevated homocysteine levels at T1 might have entered the cardiovascular rehabilitation program in an overall worst cardiovascular condition, responsible for a low performance at the six-minute walk test. Thus, an efficient rehabilitation program may have produced the larger percent increments in these patients. To support our hypothesis, we separately considered the 6MWD of HTx patients with homocysteine below or above 14 μmol/L (average level over the group). Patients with homocysteine >14 μmol/L walked a significantly lower distance than patients with homocysteine <14 μmol/L at T1 (186 ± 90 m vs. 303 ± 85 m, p < 0.01). This was not the case at T2, when the performance was similar in the two groups (374 ± 94 m vs. 422 ± 53 m, p n.s.). From a rehabilitation perspective, this finding suggests that HTx patients with a poor 6MWD evaluation and with hyperhomocysteinemia are likely to retrieve most of their lost functional capacity with a standard cardiovascular rehabilitation program, while this is less likely to occur in LVAD patients, explaining why we previously reported a greater functional performance in HTx than in LVAD patients after rehabilitation^3^.

In HTx patients we also found a positive association between IGF-1 and 6MWD% increments. HTx patients had higher IGF-1 mean levels (Fig. 3) and we mentioned the role of this growth factor in improving the nutritional status and muscle mass. It is therefore possible that IGF-1 may positively contribute to the recovery of the functional outcome in HTx patients.

As a limitation, patients were surgically treated in a single cardiac transplant centre based on the ISHLT guidelines^4^ for selecting between HTx or LVAD treatments, and results might differ if other selection criteria are followed. Furthermore, results at T2 could not be extended to patients following different types of cardio-respiratory programs and, since all our patients completed the cardiac rehabilitation (offered free of charge by the National Health System in Italy), it was not possible to separately quantify the effects of cardiac rehabilitation from the effects of a 3-week recovery period after surgery.

In conclusion, our work suggests that end stage heart failure patients treated with HTx or LVAD device may require different dietary and pharmacological approaches to better focus the cardiac rehabilitation on their needs. Care should be paid to promptly detect the early occurrence of dyslipidemia and hyperglycemia, or renal distress, in HTx patients (probably favoured by their immune suppressive therapy), modifying their diet or tailoring pharmacological treatments during rehabilitation. Furthermore, a good protein dietary support should be provided particularly to HTx patients. Finally IGF-1 seems to be an important biomarker to be monitored in LVAD patients, where it appears to be dangerously low, possibly contributing to the lower functional outcome in these patients.
